# Structural and Thermo-Mechanical Properties of Poly(ε-Caprolactone) Modified by Various Peroxide Initiators

**DOI:** 10.3390/polym11071101

**Published:** 2019-06-28

**Authors:** Marta Przybysz, Aleksander Hejna, Józef Haponiuk, Krzysztof Formela

**Affiliations:** 1 Department of Polymer Technology, Faculty of Chemistry, Gdańsk University of Technology, Gabriela Narutowicza 11/12, 80-233 Gdańsk, Poland; 2 Central Mining Institute, Department of Material Engineering, Pl. Gwarków 1, 40-166 Katowice, Poland

**Keywords:** poly(ɛ-caprolactone), reactive processing, peroxide initiators, volatile organic compounds, thermo-mechanical properties

## Abstract

The modification of poly(ε-caprolactone) (PCL) was successfully conducted during reactive processing in the presence of dicumyl peroxide (DCP) or di-(2-*tert*-butyl-peroxyisopropyl)-benzene (BIB). The peroxide initiators were applied in the various amounts of 0.5 or 1.0 pbw (part by weight) into the PCL matrix. The effects of the initiator type and its concentration on the structure and mechanical and thermal properties of PCL were investigated. To achieve a detailed and proper explication of this phenomenon, the decomposition and melting temperatures of DCP and BIB initiators were measured by differential scanning calorimetry. The conjecture of the branching or cross-linking of PCL structure via used peroxides was studied by gel fraction content measurement. Modification in the presence of BIB in PCL was found to effectively increase gel fraction. The result showed that the cross-linking of PCL started at a low content of BIB, while PCL modified by high DCP content was only partially cross-linked or branched. PCL branching and cross-linking were found to have a significant impact on the mechanical properties of PCL. However, the effect of used initiators on poly(ε-caprolactone) properties strongly depended on their structure and content. The obtained results indicated that, for the modification towards cross-linking/branching of PCL structure by using organic peroxides, the best mechanical properties were achieved for PCL modified by 0.5 pbw BIB or 1.0 pbw DCP, while the PCL modified by 1.0 pbw BIB possessed poor mechanical properties, as it was related to over cross-linking.

## 1. Introduction

The growing consciousness of the importance of environmental protection, as well as the impact of manufacturing and application of polymers on the state of the environment, have resulted in increased interest in the development of eco-friendly materials [1,2,3,4]. Attention-grabbing aliphatic polyesters, especially poly(lactic acid) (PLA), polyhydroxyalkanoates including poly(3-hydroxybutyrate) (PHB), or poly(ε-caprolactone) (PCL) are biodegradable linear polymers, obtained from both renewable and petroleum sources [5,6,7]. However, their industrial implementation is still limited due to their considerable drawbacks (e.g., low thermal stability, insufficient mechanical properties, high cost), which have led to the necessity of their further modification and reinforcing [8,9,10]. Modification of linear aliphatic polyesters leads to moderate cross-linking or branching by introducing the reactive additives, which can result in a significant increase of mechanical strength, elasticity, dimensional stability, and solvent resistance [11,12,13,14]. Hence, cross-linking is often used as an effective method in biodegradable polymer modification or compatibilization. The cross-linking of biodegradable polyesters can be performed by reactive processing [15,16,17,18,19] or by irradiation such as β or γ [20,21,22]. Both methods were widely applied to cross-linking of polyesters in the presence of a low concentration of cross-linking agents such as organic peroxides [11,23,24,25,26,27,28,29,30] and multifunctional reagents [21,31,32] or chain extenders [33,34,35,36,37]. 

The free radical initiator from organic peroxides is one of the simple methods of grafting/cross-linking of aliphatic polyesters. The formation of macro-radicals can be accomplished either via hydrogen abstraction from the methyl group/tertiary carbon or breaking of strong carbon–carbon bonds in PCL. In the peroxide-initiated modification of PCL degradation attributed to random chain, scission from the polymer backbone can occur.

The modification of PCL structure via dose irradiation and free radical initiators has been studied by various research groups. Narkis et al. [22] described the effects of the γ-irradiation dose level on the structure and physical properties of PCL. They studied doses of up to 70 Mrad, resulting in gel contents of about 35%. Yoshii et al. [38] investigated the influence of γ-irradiated in the solid state at 30 ± 55 °C, and the molten state or the super-cooled state for the change in PCL properties. Irradiation of PCL in the super-cooled state led to the highest gel content and this polymer had high heat resistance. 

Moreover, cross-linked PCL was also obtained by electron beam irradiation in the presence of polyfunctional monomer (PFM) [39]. However, few publications have been found concerning the peroxide cross-linking via reactive processing of PCL. Narkis and Wallerstein [30,40] reported that PCL could be cross-linked efficiently by usual peroxide levels and studied the rheological behavior in the pre-gelation region. Han et al. [41] investigated chemical cross-linking of PCL modified by various amounts of benzoyl peroxide (BPO).

A more advantageous method of PCL structures modification via free radical initiators is reactive processing, it represents a unique tool to manufacture biodegradable polyesters upon different types of reactive modification in a cost-effective way and leads to improving properties of the polyester. 

Nevertheless, several scientific studies focused on the cross-linking/branching of biodegradable polymers and their blends via reactive modification presented that the common vegetable oils such as linseed oil [42], soybean oil [43] and other multi-functionalized vegetable oils [44,45,46] can act as plasticizers and polymer-filler compatibilizers. The effect of plasticization using vegetable oil additives has been described for different biodegradable polyesters, including PLA [47] and PHB [48], proving that a slight decrease in the glass transition temperature occurs but toughness improves.

In this work, modification of PCL using two types of dialkyl peroxides was examined to provide a deep insight into the reaction between the PCL chains initiated by free radicals. The modification of commercial PCL through adding various amount of organic peroxide, such as dicumyl peroxide (DCP) or di-(2-*tert*-butyl-peroxyisopropyl)-benzene (BIB), was conducted aiming at improving the mechanical properties. The organic peroxides act like free radical initiators and cause branching and cross-linking of PCL structure via reactive processing. This method is an economical and simple solution because it was carried out in the melt state in the presence of small amounts of DCP or BIB, and no special purification step or complex apparatus were obligatory. The study is expected to provide information on cost-effective and eco-friendly biodegradable PCL-based material through a comprehensive evaluation and understanding of their mechanical and thermal characteristics. The peroxides possessing a relatively high hydrogen abstraction ability were selected for studying the influence of the amount of added peroxide on cross-linking/branching of PCL.

## 2. Materials and Methods 

### 2.1. Materials

The commercially available poly(ɛ-caprolactone) (PCL) (Capa 6800, Mw = 80 000 g⸱mol^−1^) was purchased from Perstorp (Malmö, Sweden). The free radical initiators dicumyl peroxide (DCP) (Peroxan DC-P+) and di-(2-*tert*-butyl-peroxyisopropyl)-benzene (BIB) (Peroxan BIB-1) were supplied by Pergan GmbH (Bocholt, Germany).

### 2.2. Preparation of Modified PCL Samples

PCL was modified by a free radical reaction initiated by two types of organic peroxides, DCP and BIB, used in different concentrations—0.5 and 1.0 pbw (part by weight). All samples were prepared in the Brabender^®^ internal mixer (type GMF 106/2) at a temperature of 170 °C, where a rotor speed was set to 100 rpm for a total time of 8 min. First the PCL polymer was premixed into a mixing chamber for 4 min, then the reactive processing in the presence of DCP or BIB followed and continued for the next 4 min. The temperature processing and the residence time were related to the cross-linking time and temperature, which were 4 min at 170 °C for DCP and 180 °C for BIB. Subsequently, prepared samples were molded to 2 mm thickness by the compression molding under pressure of 4.9 MPa at 170 °C for 1 min and then at room temperature for 5 min. The PCL polymer without organic peroxides was processed in the same conditions and was defined as reference sample.

### 2.3. Characterization of PCL Samples

#### 2.3.1. Mechanical Properties

The tensile properties of the PCL samples were measured at room temperature using a Zwick Z020 tensile tester (Ulm, Germany) equipped with a 20 kN load cell in accordance with the standard ISO 527-2. The loading rate was set to 50 mm/min. Median stress–strain curve and average tensile properties values were taken from an average of five repeats.

#### 2.3.2. Dynamic Mechanical Analysis

Dynamic mechanical properties were investigated using a dynamic mechanical analyzer DMA Q800 from TA Instruments (New Castle, DE, USA). The PCL samples in the form of strips (40 × 10 × 2 mm^3^) were measured in single cantilever mode at a constant frequency of 10Hz from −100 °C to 80 °C at a heating rate of 5 °C/min under nitrogen flow.

#### 2.3.3. Hardness

The hardness of PCL samples was determined via Shore’s method in accordance with ISO 868 at room temperature, using a Zwick 3131 hardness tester. Hardness was expressed in °ShD as a mean value of ten measurements.

#### 2.3.4. Fourier Infrared Spectroscopy

Fourier-transform infrared spectroscopy (FTIR) analysis of neat PCL and the modified PCL were recorded with a Nicolet Spectrometer IR200 from Thermo Scientific (Waltham, MA, USA). The device had an ATR attachment with a diamond crystal. Measurements were performed with 1 cm^−1^ resolution in the range from 4000 to 400 cm^−1^ and 64 scans.

#### 2.3.5. Gel Content

Gel fraction was determined by the weight remaining after dissolving the sample of a solvent such as toluene, chloroform or dichloromethane using the following Equation (1):

(1) Gel fraction (%)=WgW0×100 %

where W_0_ is the dry weight of the PCL sample and W_g_ is the dry gel component of the PCL sample after being dissolved in the solvents at room temperature for 72 h.

#### 2.3.6. Differential Scanning Calorimetry Analysis

The thermal properties of the samples were measured by differential scanning calorimetry (DSC,) carried out on a DSC 204 F1 Phoenix apparatus (from Netzsch, Selb, Germany) in the temperature range of −80–150 °C under N_2_ atmosphere at a heating rate of 15 °C/min. The melting point (*T*_m_), crystallization temperature (*T*_c_), enthalpy of cold crystallization and enthalpy of melting of each sample were investigated.

The degree of crystallization (X_c_) of PCL phase was calculated according to the following Equation (2):

(2) Xc=ΔHmΔH0×100%

where Δ*H*_m_ is the specific melting enthalpy and Δ*H*_0_ is the melting enthalpy of 100% crystalline polymer (where the melting enthalpy of 100% PCL is 136 J/g [49]).

#### 2.3.7. Thermogravimetric Analysis

The thermal stability of PCL samples (approximately 10 mg) was investigated by the thermogravimetric analysis combined with infrared spectroscopy (TGA–FTIR), which was performed using the model Q600 from TA Instruments (New Castle, DE, USA). The study was carried out in an inert gas atmosphere—nitrogen with a flow rate of 100 mL/min in the range from 25 to 750 °C with a temperature increase rate of 20 °C/min. Moreover, volatile products were directed (using heated transfer line with temperature 220 °C) to Nicolet iS10 spectrometer from Thermo Scientific (Waltham, MA, USA), in order to specify thermal degradation products during TGA analysis.

#### 2.3.8. Emission of Volatile Organic Compounds 

Volatile organic compounds (VOCs) emitted from modified PCL were determined using static headspace and gas chromatography–mass spectrometry (SHS–GC–MS). Measurements were performed using a Shimadzu GC2010 PLUS GC–MS (Kyoto, Japan) equipped with a split/splitless inlet. The GC–MS system was equipped with an AOC5000 Headspace Auto-Sampler. During analysis, the vial was transported by the injection unit from the tray to the agitator; when the sample achieved the equilibrium, the headspace sample of 2.5 mL volume was drawn from the vial and injected into the GC injector. The sampled vial was then returned by the injection unit to the tray.

## 3. Results and Discussion

### 3.1. Modification of the PCL Structure by Organic Peroxides

**Scheme 1:**
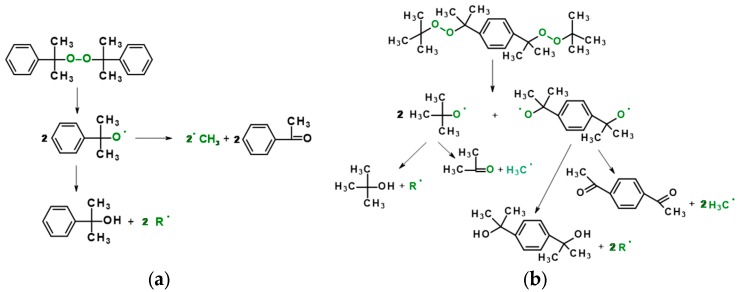
Scheme of thermal decomposition mechanism of dicumyl peroxide (DCP) (**a**) and di-(2-tert-butyl-peroxyisopropyl)-benzene (BIB) (**b**) peroxides.

The influence of type and concentration of organic peroxide on modification of PCL during reactive processing was investigated. The decomposition of the organic peroxides gives primary radicals that can be a source for secondary alkyl or aryl, which are formed by β-scission of primary radicals. In the case of dicumyl peroxide, the decomposition yields cumyloxy radicals, which are subsequently fragmented into secondary methyl radicals and acetophenone as the by-product (Scheme 1a). The secondly applied peroxide was BIB, which possesses more than one peroxy group, the decomposition yields tert-butoxy and di-(hydroxyl-isopropyl)-benzene as primary radicals and secondary methyl radicals, and diacetylebenzene, tert-butanol, and acetone as the by-products (Scheme 1b) [50].

**Figure 1:**
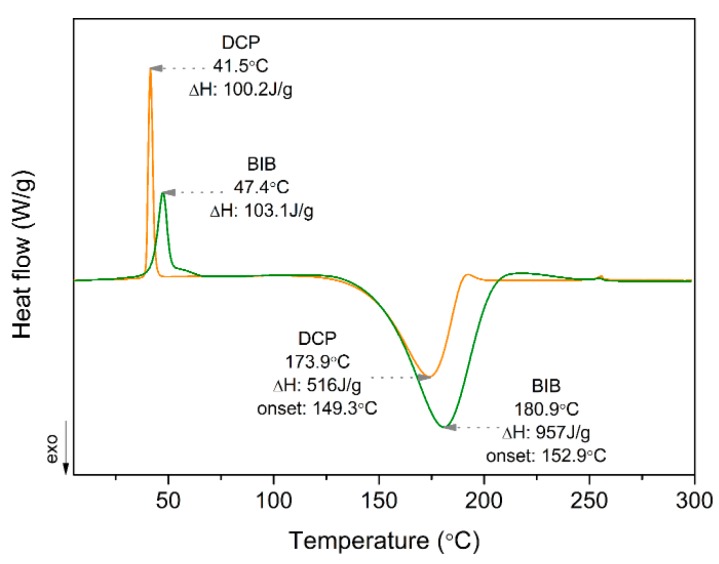
Heat flow vs. temperature for thermal decomposition of DCP and BIB peroxides.

Aiming to choose the best processing temperature, DSC analysis was conducted. DSC curves of DCP and BIB peroxides are shown in Figure 1. The melting and decomposition temperatures and melting and decomposition enthalpies of the peroxides were monitored at first heating scan. The main parameter of temperatures T_m_, T_onset_ and T_decomp._ of DCP were 41.5 °C, 149 °C and 174 °C, respectively. In the case of BIB peroxide, the temperature parameters were significantly higher, T_m_, T_onset_ and T_decomp_. were equal to 47.4 °C, 153 °C and 181 °C, respectively. Results of DSC analysis allowed to define the temperature of reactive processing for the reactive modification of PCL via free radical reaction initiated by organic peroxide.

**Scheme 2:**
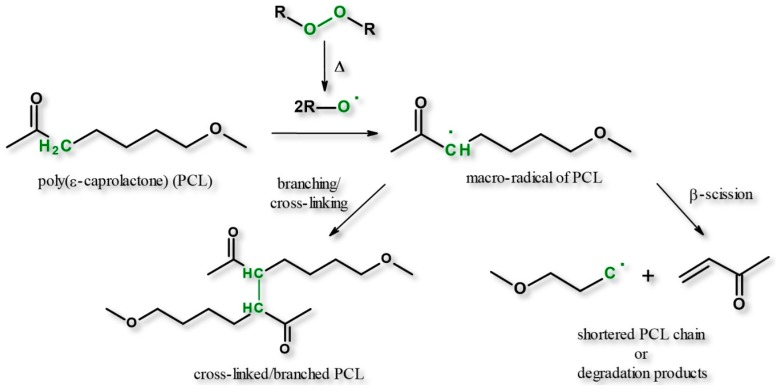
Likely reaction mechanism of the poly(ɛ-caprolactone) (PCL) in the presence of the organic peroxide.

To achieve branching or cross-linking of linear PCL structure, reactive processing initiated by organic peroxide decomposing at elevated temperature was carried out. In view of small differences of onset temperature for DCP and BIB decomposition, the modification of PCL was conducted at 170 °C. Reactive processing can be counted as a green technique because it is fast and hence has a low energy consuming process that does not involve the use of solvents and limiting the use of auxiliaries in general. The possible reaction mechanism of the PCL modified by the organic peroxide during the reactive processing is illustrated in Scheme 2. When DCP or BIB is decomposed into free radicals at elevated temperature, it could facilitate the extraction of H’s atom from the PCL chain to generate free radicals on the backbone of PCL, which is a result of propagation of the radical reaction to form a cross-linked/branched structure of PCL. The PCL generates free radicals on tertiary C atoms, which get readily stabilized during reactive processing. It is worth mentioning that there are possibilities of the formation of a strong covalent C–C chemical linkage via a recombination of PCL macro-radicals. The applying of peroxides also can lead to β-scission of chains, and result in the degradation of this polymer, which is not desired during processing.

### 3.2. Volatile Organic Compounds Emission

**Figure 2:**
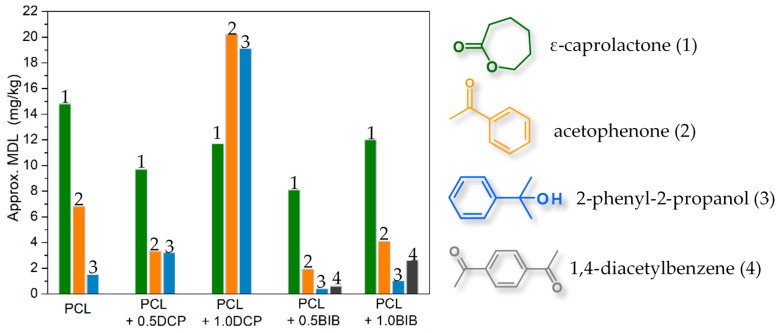
Volatile organic compounds (VOCs) emissions form PCL and PCL modified by the organic peroxides.

In order to determine the volatile organic compounds (VOCs) emission from the samples, the static headspace and gas chromatography–mass spectrometry analysis was performed. The results are displayed in Figure 2. The VOCs measurements revealed that ε-caprolactone, acetophenone and 2-phenyl-2-propanol were the main volatile organic compounds (VOCs) for all PCL samples, and additionally 1,4-diacetylbenzene for PCL plus BIB. The growth in the VOC amount was related to by-products of peroxides decomposition and low molecular products of free radical reactions, e.g., acetophenone, methane or 2-phenylo-2-propanolfrom DCP and diacetylobenzene, tert-butanol, acetone or di-(2-hydroxyisopropyl)benzene in case of BIB, as it was mentioned above (Scheme 1). In the case of PCL modified by BIB peroxide, the amount of the VOCs was lower than for PCL samples with added DCP. It is related to the reactivity of used peroxides. The modification of PCL in the presence of BIB occurs more dynamically, and just after the adding of peroxide the emission of VOCs during reactive processing occurs.

### 3.3. Thermal Stability

The thermal stability of PCL modified by organic peroxides was determined by thermogravimetric analysis. The temperatures corresponding to the –2%, –5%, –10% and –50% mass loss for PCL are essential for evaluating their thermal stability and are summarized in Table 1. The employing of small amounts of DCP and BIB has an insignificant effect on the thermal stability of the PCL; the T_–2%_ slightly decreased to 359 °C and 364 °C, respectively, while T_–2%_ was 375 °C for the reference PCL. The similar influence of peroxides added on mass loss was observed for PCL modified by 1.0 DCP and 0.5 BIB. It confirms that the applying of 1.0 DCP gives the same effect as for 0.5 BIB. The biggest difference in thermal parameters was seen for PCL modified by 1.0 BIB, which is related to the higher reactivity of that peroxide.

**Figure 3:**
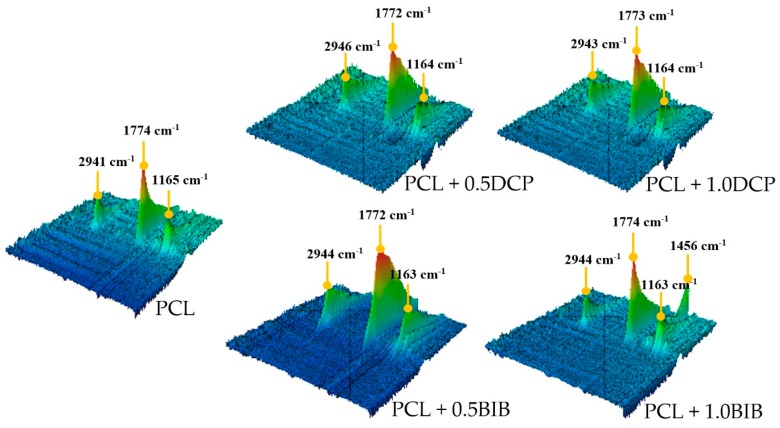
Three-dimensional image of TGA–FTIR analysis for PCL and PCL modified by DCP and BIB.

FTIR spectra of evolved gases from thermal degradation of PCL samples are presented in Figure 3. As it can be seen, the spectrum of the evolved gases exhibits bands in the range of 2944–2872 cm^−1^ related to C-H stretching of methylene groups and a band at 1164 cm^−1^ associated with C-O stretching. Besides, an intense band in the carbonyl region was also detected. A closer inspection of this band demonstrated that the carbonyl peak is composed of three overlapped absorptions located at 1772, 1756 and 1736 cm^−1^. The peak at 1772 cm^−1^ can be associated with carboxylic acids in gaseous phase and, in fact, some authors have related this absorption to compounds such as hexenoic acid [51,52,53] and/or hexanoic acid (caproic acid) [54]. While the band at 1736 cm^−1^ has been related to the emission of ε-caprolactone [55], this signal can be associated with ester groups of small chain fragments of PCL. The band related to ε-caprolactone ought to be located at 1756 cm^−1^ as the internal tension in the cyclic structures increases the carbonyl-stretching absorption frequency in comparison with the acyclic ones. However, we could not confirm that the thermal degradation of PCL proceeded in a one-stage degradation mechanism based on the above analysis.

**Scheme 3:**
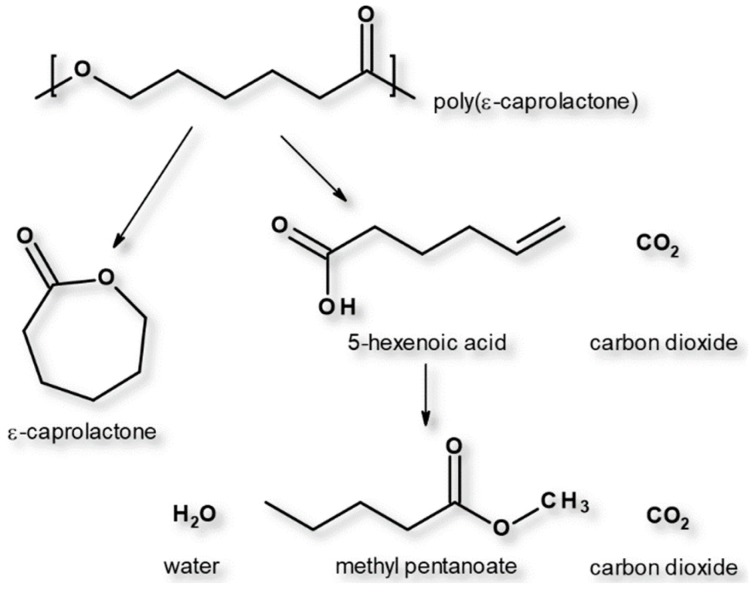
The thermal degradation mechanism of PCL.

Literature data [52,53] indicated that thermal degradation of PCL occurs in two steps. The first way involves a statistical rupture of the polyester chains yielding H_2_O, CO_2_ and hexenoic, while in the second, ε-caprolactone is evolved as results of an unzipping depolymerization process [56]. In our case, results suggest that both processes occur simultaneously, with the emission of small chain fragments of PCL being the later event. Moreover, the CO_2_ emission is produced (see bands at 2358 and 672 cm^−1^) during this process, but its amount is almost invisible. The complete thermal degradation mechanism of PCL was summarized in Scheme 3 [51].

### 3.4. Thermal Properties

**Figure 4:**
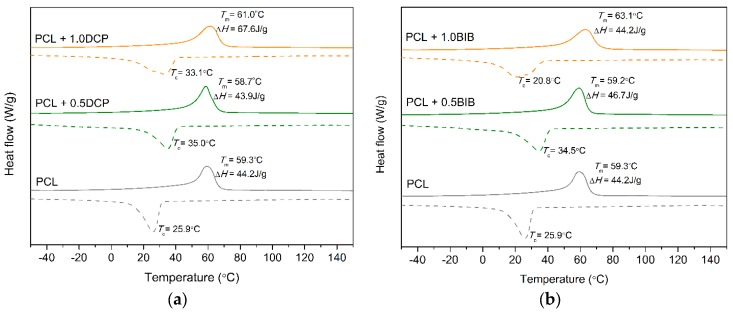
DSC thermograms for PCL modified by DCP (**a**) and PCL modified by BIB (**b**).

The thermal properties of the PCL modified by organic peroxides were investigated by differential scanning calorimetry (DSC) measurements. DSC analysis was carried out to understand the crystallization and melting behavior of modified PCL. The obtained second heating and cooling curves of PCL modified by DCP and BIB are depicted in Figure 4a,b, respectively. The corresponding thermal parameters are listed in Table 2. All of the studied samples exhibit evident melting (*T*_m_) and crystallization (*T*_c_) temperatures, the corresponding for PCL are approximately 60 °C (*T*_m_) and 30 °C (*T*_c_). After the addition of organic peroxides to PCL, the melting temperatures did not shift significantly. The influence of DCP, as well as BIB’s, on the melting temperatures of PCL is minimal. It is noted that the cold crystallization of PCL modified by organic peroxide tends to shift to higher temperatures in comparison to unmodified PCL chains. The percentage crystallinity (X_c_) values of PCL sample domains are determined, and their values are summarized in Table 2. The addition of organic peroxides via reactive processing to the neat PCL results in marginal increase or no appreciable change in X_c_ of modified PCL. In case of the higher amount of DCP into PCL, the increasing of X_c_ can be related to the crystallinity of short chains. Moreover, the melt state modification of polymers capable of crystallization affects only the linear segments of the polymer chains. Additionally, the entangled linear segments often increase the degree of crystallinity in polymers that are exposed to degradation.

### 3.5. Dynamic Mechanical Analysis

**Figure 5:**
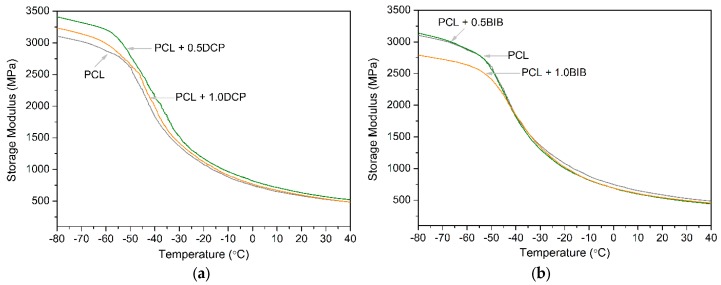
Storage Modulus for PCL modified by DCP (**a**) and PCL modified by BIB (**b**).

**Figure 6:**
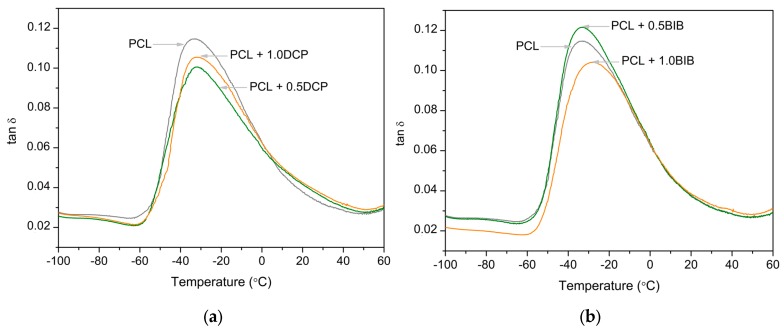
Loss angle (tanδ) for PCL modified by DCP (**a**) and PCL modified by BIB (**b**).

The results of one point bending test of PCL unmodified and modified by peroxides investigated via dynamic mechanical analysis (DMA) are shown in Figure 5 and Figure 6. Dependence of the storage modulus (E’) and tanδ at 25 °C for PCL samples are listed in Table 3. Figure 5a,b shows the variation of the storage modulus (E’) of neat PCL and modified PCL samples as a function of temperature. The curves of modified PCL samples have the same shape as neat PCL. As shown, the storage modules for the PCL modified by DCP are higher than those of neat PCL over the whole temperature range examined here. The effects of the organic peroxides on the dynamic mechanical properties of modified PCL were investigated. The dynamic mechanical results show a significant increase of E’ of composites modified by 0.5 pbw of DCP, while higher content of DCP leads to a slight decrease. The reason for this increase can be related with branching of the PCL structures in case of modification with DCP. For PCL modified by 1.0 pbw of BIB, the E’ was substantially decreased.

Figure 6a,b shows the results of tanδ for unmodified PCL and modified PCL samples. For neat PCL and modified PCL only one glass transition was seen from the tanδ peak, and the transition temperature did not shift significantly. The tanδ transitioned to higher temperatures only for PCL modified by 1.0 pbw of BIB. Likewise, storage modulus (E’) dropped only in case of the same sample. This could occur due to the possibility of degradation of PCL in the presence of high BIB content and β-scission of chains during processing and then cross-linking of shorter PCL chains.

### 3.6. Static Mechanical Properties

**Figure 7:**
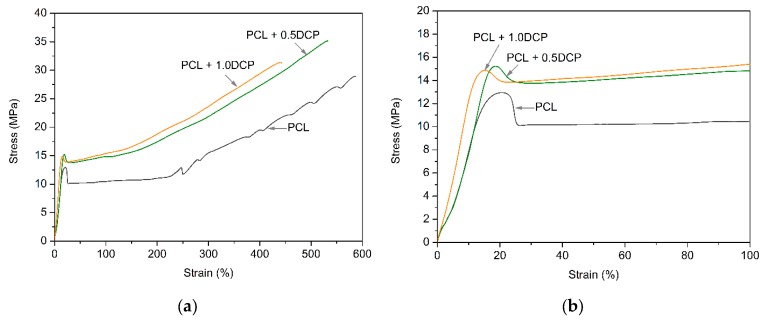
The stress–strain curves for PCL modified by DCP peroxide.

**Figure 8:**
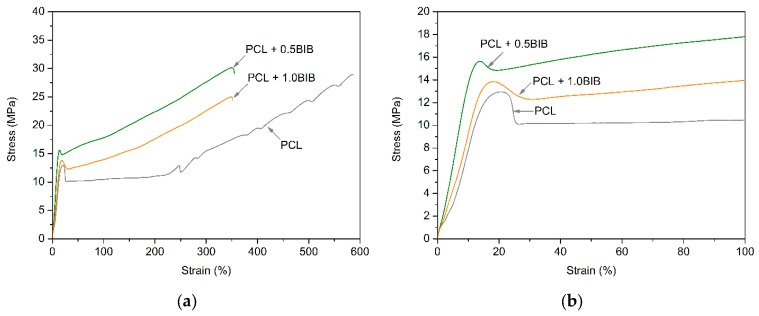
The stress–strain curves for PCL modified by BIB peroxide.

Results of the tensile strength, elongation at break, and hardness of studied PCL are listed in Table 4. An apparent yielding is evident in the stress–strain curves of all of the PCL modified by DCP and modified by BIB shown in Figure 7 and Figure 8, respectively. As it can be observed, the tensile strength of PCL modified by DCP and 0.5 pbw of BIB noticeably increased, while the elongation at break significantly decreased for those samples. It confirms that free radicals enhance interactions between PCL chains, toward branching or cross-linking.

Improvement of tensile strength, elongation at break, and hardness confirms that introduction of DCP and BIB peroxide positively influences on mechanical properties of PCL. The hardness of PCL modified by organic peroxides increased from 51°Sh D to 53°Sh D and 52°Sh D for the unmodified polymer with added DCP and with added BIB, respectively. The effect was negligible for PCL modified by 1.0 pbw of BIB, both in hardness and tensile strength. Furthermore, application of a higher amount of the BIB peroxide leads to deterioration of the elongation at break much more than of the DCP peroxide. The mechanical properties of PCL, PCL/DCP, and PCL/BIB are summarized in Table 3. Unmodified PCL characterizes quite good tensile strength and high elongation at break, characteristic features of a ductile material. Modification of PCL using a moderate amount of peroxide leads to improvement of tensile strength and hardness. In this case, the appropriate peroxide concentration was 0.5 pbw, for DCP as well as BIB. However, tensile tests indicated that tensile strength and elongation at break decreased with increasing peroxide content. The values of tensile strength at break decreased from 35.7 ± 1.9 MPa to 29.1 ± 1.7 MPa and from 36.7 ± 2.2 MPa to 23.1 ± 1.4 MPa for DCP and BIB peroxide, respectively. In the comparison of the tensile strength of unmodified PCL to PCL modified by 0.5 pbw, peroxide improved. In case of the modification of PCL in the presence of 1.0 BIB, the tensile strength, elongation at break, significantly decreased, as it was related to over cross-linking (the gel fraction in this sample was above 50%—see Table 6) affecting the higher brittleness of the material. Similar observations were pointed out by Yang et al. [8], who evaluated thermo-mechanical properties of cross-linked poly(lactic acid).

### 3.7. Fourier-Transform Infrared Analysis

**Figure 9:**
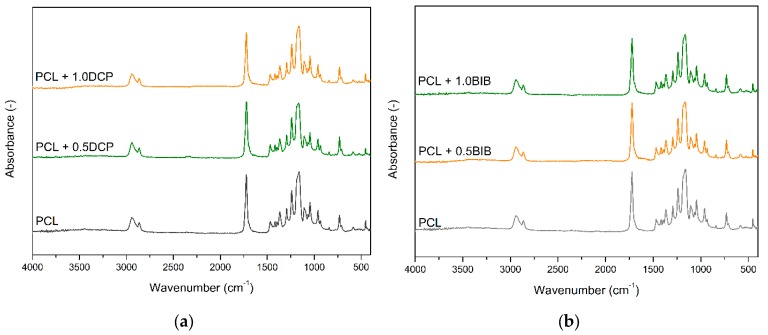
FTIR absorption spectra of PCL modified by DCP (**a**) and PCL modified by BIB (**b**).

The Fourier-transform infrared (FTIR) technique is used to identify the structural modification taking place during the chemical reactions among PCL in the presence of DCP and BIB, as presented in Figure 9a,b, respectively. FTIR was applied to investigate the chemical structure of neat PCL and PCL modified by organic peroxides. Figure 9 shows the absorption spectra of PCL and modified PCL in the total wavelength range of 4000–400 cm^−1^. Regarding the spectra of PCL samples, we can easily identify two peaks at 2942 and 2863 cm^–1^ which indicated the presence of C-H stretching, strong bands such as the carbonyl stretching mode and C-O bending around 1722 cm^−1^ and 1140 cm^–1^ [57]. The characteristic strongest bands and their assignments with all of the PCL samples are listed in Table 5. Introduction of a small amount of organic peroxides to the PCL matrix did not demonstrate significant changes in the structure, which we can observe by using FTIR spectroscopy.

### 3.8. Gel Fraction

As mentioned in the scheme of the possible reaction mechanism (Scheme 2), the PCL in the presence of organic peroxide shows a complex type of reaction through the formation of a cross-linking structure as well as branching in presence of DCP and BIB. The gel content of the neat PCL and samples modified by organic peroxides are presented in Table 6. It is found that the gel fraction of PCL modified by BIB peroxide is significantly higher in comparison to that of PCL modified by DCP. It clearly showed that the cross-linking of PCL starts at the low content of BIB peroxide, while the PCL modified by 0.5 DCP was completely soluble in all used solvents. On the other hand, the sample with 1.0 pbw DCP peroxide was partially soluble, therefore it was impossible to measure actual gel fraction content. The reason behind such different behavior for the used peroxides is the development of cross-linking of the PCL modified by BIB, which resulted in high gel content. The gel fraction of PCL increases with increasing BIB content. The gel fraction is approximately 38% and 53% by adding 0.5 pbw and 1.0 pbw of BIB, respectively, both at toluene and dichloromethane. Nevertheless, although the amounts of added peroxide were doubled, the gel fraction content increase was not twice as high, as no linear correlation was observed. This suggests that a cross-linking of shorter PCL chains might have occurred. The DCP is a less reactive peroxide than BIB; it is related in chemical structure. Therefore, the mechanical properties of PCL cross-linked by 1.0BIB were lowest, which resulted in over cross-linking of PCL. These results support that the modification of PCL structure depended on the type of peroxides and their amount.

## 4. Conclusions

The organic initiators such as di-(2-*tert*-butyl-peroxyisopropyl)-benzene (BIB) and dicumyl peroxide (DCP) introduced to linear poly(ε-caprolactone) (PCL) lead to modification of the polymer structure. This study establishes an easy-to-implement reactive processing approach in the melt state to achieve changes of PCL architecture towards branching and/or cross-linking. The mechanical properties and gel fraction showed that modification with DCP peroxide leads to a branching of PCL structure, while BIB peroxide proved to be an effective cross-linking agent for PCL. However, the modification of PCL using organic peroxide is a complicated process, where all reactions, such as branching and/or cross-linking but likewise the degradation of PCL, can occur. Specific investigation of modified PCL showed that the higher BIB content can lead to degradation and over cross-linking of PCL; this was confirmed by the decline of mechanical, thermal properties and not linear increase of gel fraction.

In summary, a simple, effective, economically balanced strategy to tune the thermo-mechanical behavior of a commercially available compound has been exhibited. Reactive modifications were performed in the melt state by addition of low amounts of peroxide, using standard equipment and without any complex final purification steps, and could be easily modulated by the amount of added peroxide under solvent-free conditions. There are potential applications of branched/cross-linked PCL due to the degradation time, which can be extended, in the automotive and gardening industries.

## Figures and Tables

**Table 1:** Thermal degradation parameters of PCL and PCL modified by the peroxides.

Samples	Weight Ratios	T_–2%_ (°C)	T_–5%_ (°C)	T_–10%_ (°C)	T_–50%_ (°C)	R_700°C_ (%)
PCL	100	375	390	398	422	0.7
PCL/DCP	100/0.5	359	378	390	420	1.6
	100/1.0	367	387	397	421	1.4
PCL/BIB	100/0.5	364	383	395	429	2.0
	100/1.0	321	363	381	415	1.0

T_–2%_, T_–5%_, T_–10%_, T_–50%_ are the temperatures corresponding to the –2%, –5%, –10% and –50% mass loss, respectively; and R_700_ is the residual weight at 700 °C.

**Table 2:** DSC parameters (second heating) of PCL modified by DCP and BIB.

Samples	Weight Ratios	*T*_m(PCL)_ (°C)	Δ*H*_m(PCL)_ (J/g)	*T*_c(PCL)_ (°C)	Δ*H*_c(PCL)_ (J/g)	X_c(PCL)_ (%)
PCL	100/0	59.3	44.2	25.9	54.7	32.5
PCL/DCP	100/0.5	58.7	43.9	35.0	45.5	32.3
	100/1.0	61.0	67.6	33.1	50.0	49.7
PCL/BIB	100/0.5	59.2	46.7	34.5	48.3	34.3
	100/1.0	63.1	44.2	20.8	56.0	32.5

**Table 3:** The dynamic mechanical analysis parameters of PCL and PCL modified by peroxides.

Samples	Weight Ratios	tan δ_max_ (°C)	E’_25°C_ (MPa)
PCL	100/0	−33.5	512
PCL/DCP	100/0.5	−32.2	599
	100/1.0	−31.2	563
PCL/BIB	100/0.5	−33.0	507
	100/1.0	−28.5	517

**Table 4:** The mechanical properties of PCL and PCL modified by peroxides.

Samples	Weight Ratios	Young’s Modulus (MPa)	Stress of Yield (MPa)	Stress at Break (MPa)	Elongation at Break (%)	Hardness (°Sh D)
PCL	100	149 ± 25	13.1 ± 0.2	30.0 ± 1.2	611 ± 25	51.6 ± 0.7
PCL/DCP	100/0.5	183 ± 16	15.0 ± 0.8	35.7 ± 1.9	521 ± 23	53.0 ± 0.5
	100/1.0	169 ± 11	14.6 ± 0.5	29.1 ±1.7	430 ± 9	53.3 ± 0.4
PCL/BIB	100/0.5	143 ± 12	15.4 ± 0.5	36.7 ± 2.2	471 ± 18	52.9 ± 0.9
	100/1.0	160 ± 13	13.4 ± 0.6	23.1 ± 1.4	372 ± 47	52.1 ± 0.2

**Table 5:** Characteristic FTIR peak of the neat PCL and modified by peroxides.

Wavenumber Assignments	Neat PCL	PCL + DCP		PCL + BIB	
		0.5	1.0	0.5	1.0
asymmetric CH_2_ stretching ν_as_(CH_2_)	2942	2944	2941	2941	2942
symmetric CH_2_ stretching ν_s_(CH_2_)	2863	2863	2862	2863	2863
carbonyl stretching ν(C=O)	1722	1722	1721	1722	1722
asymmetric COC stretching ν_as_(COC)	1240	1239	1238	1240	1240
OC-O stretching ν(OC-O)	1187	1184	1184	1187	1187
symmetric COC stretching ν_s_(COC)	1160	1166	1162	1164	1165

**Table 6:** Gel fraction content of modified PCL in the presence of the organic peroxides.

	Samples Code	PCL100	PCL + 0.5DCP	PCL + 1.0DCP	PCL + 0.5BIB (%)	PCL + 1.0BIB (%)
Solvent						
toluene		*soluble*	*soluble*	*partially soluble*	37.9 ± 0.8	53.5 ± 0.9
dichloromethane		*soluble*	*soluble*	*partially soluble*	37.7 ± 0.3	53.0 ± 0.4
chloroform		*soluble*	*soluble*	*partially soluble*	41.1 ± 0.6	50.0 ± 0.7
